# Advances in diagnostic methods for detection of bloodstream pathogens and antibiotic resistance determinants

**DOI:** 10.3389/fcimb.2025.1725920

**Published:** 2025-12-09

**Authors:** Sherry A. Dunbar, Martha Benavides, Christopher Gardner

**Affiliations:** Diasorin, Austin, TX, United States

**Keywords:** bloodstream infection (BSI), sepsis - diagnostics, antimicrobial resistance (AMR), molecular diagnostics, antimicrobial stewardship (AMS), diagnostic stewardship

## Abstract

This Perspective highlights the growing threat of multidrug-resistant bacteria in bloodstream infections and explores the role of various diagnostic technologies in the early identification of bacterial pathogens and antibiotic resistance genes. We review current diagnostic approaches and their applications in surveillance, infection control, and antimicrobial stewardship. Rapid detection of bloodstream pathogens and resistant organisms enables clinicians to promptly tailor treatment, improve patient outcomes, reduce complications, and shorten hospital stays. Emerging innovations in machine learning, artificial intelligence, whole genome sequencing, and point-of-care molecular diagnostics hold great promise for enhancing the detection and management of these serious infections.

Multidrug-resistant organisms (MDROs) in bloodstream infections (BSIs) present a dangerous global threat. They drive higher morbidity, mortality, and escalate healthcare costs due to failed empiric therapy, delay in effective therapy, and prolonged hospitalizations. The 2016 O’Neill report on antimicrobial resistance (AMR) projected 10 million annual deaths globally and a cumulative economic cost of $100 trillion by 2025 if this crisis is not addressed (Price, 2016). In the US, annual economic burden was estimated at $20 billion in healthcare costs and $35 billion in productivity loss due to AMR infections. Between 2019 and 2021, sepsis-related deaths from infectious causes increased by 86.4%, accounting for 31.5% of all global deaths (Global, regional, and national sepsis incidence and mortality, 1990-2021: a systematic analysis, 2025). In addition to its clinical impact, MDRO-related sepsis drives up healthcare costs through failed empiric therapies, prolonged hospital stays, and increased resource utilization. By 2023, it was reported that 1 in 6 bacterial infections worldwide were resistant to standard antibiotics (WHO Global Antimicrobial Resistance and Use Surveillance System (GLASS), 2025). These resistant pathogens significantly contribute to the burden of sepsis, which affects over 30 million people globally each year (Fleischmann et al., 2016).

Advanced diagnostics and innovative techniques play a crucial role in early detection of MDROs, enabling timely initiation of effective therapy, supporting infection control and surveillance, and guiding patient management and antimicrobial stewardship (ASP). Rapid, accurate results help limit overuse of broad-spectrum antimicrobial agents and are crucial to effective stewardship and outbreak control.

Conventional blood culture is still considered the gold standard for diagnosing BSIs but is slow—requiring 6 to 60 hours for pathogen identification (Afshari et al., 2012; Yo et al., 2022). This time lag often forces empiric broad-spectrum antibiotic use. Standard phenotypic antimicrobial susceptibility testing (AST), though essential for guiding therapy, typically takes an additional 18–24 hours post-culture, limiting its ability (Hattab et al., 2024). Developing rapid AST technologies is a growing priority in the United States, driven by the need to combat antimicrobial resistance (AMR) and improve outcomes in bloodstream infections. Adopting rapid AST technologies can be transformative but it is not without hurdles, such us, the operational challenges of integrating into existing workflows, staff training, sample limitations, as well as financial and resource barriers, and stakeholder buy-in (Roth et al., 2022). Recent developments such as deep-learning–based optical AST systems can deliver susceptibility results in approximately 6 to 7 hours using neural networks and standard workflows, promising faster and more actionable data (Brown et al., 2020; Roth et al., 2022). These technologies are promising but are still limited in several respects, including data modeled only in controlled settings using small sample sizes and narrow sets of species, heterogenous/mixed populations, polymicrobial infections, and lack of robust testing to cover variations in sample preparation, instrumentation, and imaging conditions (Zagajewski et al., 2023; Liao et al., 2025).

Matrix-assisted laser desorption/ionization time-of-flight mass spectrometry (MALDI-TOF MS) has transformed conventional microbiology by providing species-level identification in minutes from positive blood cultures, dramatically shortening turnaround time and improving clinical outcomes. The review by Yo et al., describes how MS methods have resulted in a 23% reduction in BSI mortality, about 5 hours less time to effective therapy, a 23-hour reduction in time to pathogen identification, ~0.73-day shorter hospital stay, and an average $4,140 direct cost saving per case (Yo et al., 2022). Notably, the combination of MALDI-TOF with AST and ASP intervention further improves outcomes (Nomura et al., 2020; Dunbar et al., 2022; Liborio et al., 2024). Nonetheless, MALDI-TOF has limitations, particularly with polymicrobial samples, some fungi, and resistance detection (Nomura et al., 2020; Hosoda et al., 2025).

Molecular assays, including multiplex PCR panels, are capable of detecting specific pathogens and key resistance genes directly from positive blood cultures, greatly reducing turnaround time compared to culture (Dunbar et al., 2022; Samuel, 2023; Liborio et al., 2024). These methods can be differentiated by whether they utilize direct detection (e.g., PCR performed directly from blood) or amplification-based methods including qPCR, multiplex panels with nucleic acid amplification, or signal amplification of nucleic acids present in positive blood cultures. Approaches that analyze whole blood directly suffer from lower sensitivity and at present cannot fully replace blood culture (Samuel, 2023; Rapszky et al., 2025). Rapid molecular panels are expanding to detect more pathogens and a more comprehensive array of AMR genes, such as extended-spectrum beta-lactamases (ESBLs) and carbapenemases, allowing quicker recognition of MDROs which is critical for both ASP and infection control (Briggs et al., 2021; Liborio et al., 2024).

The introduction of rapid PCR-based multiplex diagnostics, combined with the establishment of core elements for antimicrobial stewardship programs (ASPs), has transformed infectious disease management (Serapide et al., 2025). In clinical practice, faster pathogen identification reduces diagnostic uncertainty and enables more targeted antibiotic prescribing. By leveraging rapid diagnostics, ASPs help guide clinicians away from broad-spectrum empiric therapy and toward precise, pathogen-directed treatment, and the combination of rapid diagnostics with ASPs has been shown to significantly lower mortality, reduce hospital stays, and lower readmission rates (Peri et al., 2024).

Recent innovations, such as whole genome sequencing (WGS) and metagenomic next-generation sequencing (mNGS) allow direct identification of pathogens and resistance genes directly from isolates or positive blood cultures or clinical specimens, respectively, providing precise genotypic data useful for tracking outbreaks and comprehensive resistance profiling (Peterson et al., 2023; Di Pilato et al., 2025). WGS is a standard method in research, public health surveillance, and confirmation of isolates where core-genome sequence typing (cgST or cgMLST/LIN) or SNP-based approaches can then be used to assess genetic relatedness (Duval et al., 2023; Lakicevic et al., 2023). Alternatively, mNGS is emerging as a rapid, culture-independent diagnostic tool and can be especially useful when early identification and resistance prediction are critical (Shinge et al., 2025). However, implementation and utilization of these technologies can be challenging due to high cost, complex bioinformatics, human DNA interference, contamination risk, reduced sensitivity in low-burden samples, laboratory validation requirements, and longer turnaround time. Although, PCR and/or sequencing approaches can provide rapid identification of AMR genes, it cannot confirm gene expression or phenotype and thus interpretation can be challenging.

Advanced diagnostics are extremely important for surveillance as molecular and sequencing technologies allow timely detection of emerging resistance within healthcare facilities, with enhanced data quality and public health reporting. Rapid diagnostics help identify clusters of MDRO infections before widespread dissemination to identify outbreaks early. WGS and metagenomics assist in linking clinical cases to transmission pathways, refining infection control strategies, and can integrate with epidemiological data (Di Pilato et al., 2025). Faster diagnostics enable targeted therapy, reducing the time and need for empirical antibiotic use and lowering selective pressure for emerging resistance. Rapid identification of BSIs aids appropriate cohorting, isolation, and de-escalation strategies to guide targeted interventions. Diagnostics informed by surveillance support a swift response to MDRO outbreaks and augment outbreak preparedness. Rapid pathogen identification, especially combined with ASP, directly correlates with better patient outcomes (Peri et al., 2024). Faster genotypic data for antimicrobial resistance genes allows tailored treatment choices based on the organism, resistance genes, and patient considerations.

In the future, MDRO diagnostics will include point-of-care molecular testing. Portable and rapid molecular platforms using CRISPR technology are in development that will be capable of delivering the diagnosis at the bedside (Deb, 2025). Rapid AST driven by artificial intelligence (AI) and machine learning (ML) will speed results and integrate laboratory and clinical workflows (Brown et al., 2020; Liao et al., 2025). Further advancements in sequencing technologies will provide faster real-time sequencing for identification and resistance, leading to rapid WGS-based diagnostics (Di Pilato et al., 2025). Various recent studies have described CRISPR-Cas systems for rapid detection and identification of bloodstream pathogens and AMR genes (Wu et al., 2021; Lim et al., 2025). Realistically, it could be implemented for species identification from positive blood cultures within the next few years as it detects pathogen-specific DNA or RNA. It may potentially be used as an adjunct in AST in the longer term but, similar to other molecular methods, it detects genetic sequences rather than the phenotypic response of an organism to antimicrobial exposure (Dara et al., 2025). CRISPR assays have not yet become commercially-available or received regulatory clearance for diagnosis of BSI pathogens or BSI AST.

There will be challenges to access and adopt rapid diagnostics, even for the most serious infections like BSIs and MDROs. High up-front costs for MALDI-TOF, molecular panels, or sequencing inhibits widespread use. Healthcare systems must consider the financial investment and impact to hospital budgets contrasted with the long-term savings. Insurance and reimbursement may be difficult as many payers fall behind in reimbursing advanced diagnostic services. Specialized instrumentation and adequate staffing will require appropriate laboratory infrastructure and technical expertise. Regional and global inequalities may lead to limited access in resource limited settings. Regulatory hurdles may slow approval of new diagnostic methods and absence of standardized universal protocols slows adoption. Robust real-world evidence will be required for providers for clinical integration. Finally, simplified workflows will be essential for laboratories to be able to validate new and advanced methods and to confidently implement these technologies effectively.

As rapid diagnostic platforms become more sophisticated and widespread, an integrated approach that combines diagnostic stewardship with antimicrobial stewardship is essential to effectively address the global challenge of AMR. These two disciplines operate in tandem: diagnostic stewardship ensures that testing is purposeful and results are clinically meaningful, while antimicrobial stewardship translates those results into timely, evidence-based treatment decisions (Ku et al., 2023). Together, they create a dynamic cycle that strengthens clinical decision-making and curbs inappropriate antibiotic use. Advancing more effective antimicrobial therapy will depend on deploying these technologies thoughtfully and synergistically with integrated stewardship models that connect precision laboratory diagnostics with bedside patient care. As diagnostics continue to evolve, so too must these frameworks that guide their use.

## Data Availability

The original contributions presented in the study are included in the article/supplementary material. Further inquiries can be directed to the corresponding author.
